# Augmentation of Pedicle Screws Using Bone Grafting in Patients with Spinal Osteoporosis

**DOI:** 10.17691/stm2021.13.5.01

**Published:** 2021-10-29

**Authors:** A.E. Bokov, A.A. Bulkin, I.S. Bratsev, S.Ya. Kalinina, S.G. Mlyavykh, D.G. Anderson

**Affiliations:** Head of the Department of Oncology and Neurosurgery, Institute of Traumatology and Orthopedics Privolzhsky Research Medical University, 10/1 Minin and Pozharsky Square, Nizhny Novgorod, 603005, Russia; Staff Neurosurgeon, Department of Oncology and Neurosurgery, Institute of Traumatology and Orthopedics Privolzhsky Research Medical University, 10/1 Minin and Pozharsky Square, Nizhny Novgorod, 603005, Russia; Staff Neurosurgeon, Department of Oncology and Neurosurgery, Institute of Traumatology and Orthopedics Privolzhsky Research Medical University, 10/1 Minin and Pozharsky Square, Nizhny Novgorod, 603005, Russia; Staff Neurosurgeon, Department of Oncology and Neurosurgery, Institute of Traumatology and Orthopedics Privolzhsky Research Medical University, 10/1 Minin and Pozharsky Square, Nizhny Novgorod, 603005, Russia; Director of the Institute of Traumatology and Orthopedics Privolzhsky Research Medical University, 10/1 Minin and Pozharsky Square, Nizhny Novgorod, 603005, Russia; Professor, Departments of Orthopaedic and Neurological Surgery; Clinical Director of the Spine Section, Orthopaedic Research Laboratory Thomas Jefferson University, 130 S., 9^th^ St., Philadelphia, PA, 19107, USA

**Keywords:** pedicle screw fixation, pedicle screw instability, vertebral augmentation, bone cement, low bone density, polymethyl methacrylate

## Abstract

**The aim of the study** was to develop a new method of vertebral augmentation based on autologous and allogeneic bone chips to be used in pedicle screw fixation and to compare this method with the technique based on polymethyl methacrylate (PMMA).

**Materials and Methods:**

This prospective non-randomized study included 164 patients with degenerative pathologies or traumatic injuries of the lumbar spine and transitional thoracolumbar segments; 153 of the operated patients were followed up for 18 months. In these patients, radiodensity of the cancellous bone tissue was below 110 HU by the Hounsfield scale. Patients with degenerative spinal disorders underwent pedicle screw fixation using transforaminal interbody fusion; patients with traumatic spinal injuries underwent intermediate pedicle screw fixation, and those with a loss of vertebral body height by >50% underwent anterior fusion.

The patients were divided into three groups: in group 1 (n=39), bone tissue augmentation was performed using PMMA; in group 2 (n=21), augmentation was done with bone chips; in group 3 (n=93), no augmentation was performed (control group). The follow-up period was 12 months; cases with fixator breakage or loosening were recorded.

**Results:**

After augmentation with PMMA, 11 cases (28.2%) of fixator destabilization were detected. With bone chips, fixator instability developed in 2 patients (9.5%) only, whereas in patients operated without augmentation, the instability was observed in 43 cases (46.2%). With PMMA augmentation, the incidence rate of fixator destabilization did not significantly differ from that in the control group (p=0.0801), while the use of bone chips resulted in a statistically significant decrease of this index compared to the control group (p=0.0023). A logistic regression analysis confirmed the superiority of the developed method over the PMMA-based vertebral augmentation.

**Conclusion:**

The use of bone chips for vertebral augmentation provides a statistically significant decrease in the incidence of pedicle screw fixator destabilization in the post-operative period. By reducing the risk of proximal loosening and eliminating the risk of bone cement drainage into the spinal canal and vascular bed, the proposed method may become especially effective in patients with impaired bone density.

## Introduction

The worldwide incidence of degenerative spine diseases and high-energy spinal injuries is on the rise [1, 2]. Degenerative stenosis of the spinal canal with segment instability and unstable spinal trauma are indications for stabilizing interventions using pedicle screw fixation and various types of spinal fusion known to provide clinically significant outcomes.

One of the most common complications of rigid spinal fixation is the destabilization of pedicle screw fixator, which occurs in 4–20% of operated patients and can exceed 50% in patients with impaired bone density [3–5]. In order to increase the stability of pedicle screw fixation, bone tissue augmentation using bone cement made of calcium phosphate and polymethyl methacrylate (PMMA) was proposed [6]. As reported, PMMA-based augmentation provided the strongest fixation; however, this method has significant drawbacks associated with using liquid cement [6]. Among such complications, there is a cement leakage into the spinal canal which may cause compression of the spinal cord and roots. If this cement gets into the vascular bed, pulmonary embolism may ensue. A syndrome of cement implantation, which can lead to acute heart failure and even sudden death in the early postoperative period, is also described [7–9]. These problems associated with PMMA-based augmentation emphasize the need to increase the strength of pedicle screw fixation without using bone cement.

**The aim of the study** was to develop a method of vertebral augmentation based on autologous and allogeneic bone chips to be used in pedicle screw fixation and to compare this method with the technique based on polymethyl methacrylate.

## Materials and Methods

This longitudinal prospective non-randomized study included 164 patients with degenerative spine disorders and unstable traumatic injuries (65 men, 99 women; mean age 65.2 (31.0–82.0) years; 153 patients (93.3%) were followed up for 18 months.

This study was conducted in accordance with the Helsinki Declaration (2013) and approved by the Ethics Committee of the Privolzhsky Research Medical University (Nizhny Novgorod, Russia). Informed consent was obtained from each patient.

During preoperative examination, the patients were presented with signs of spinal osteoporosis; the diagnosis was verified by measuring the radiodensity of the cancellous vertebral bone tissue, which was <110 HU on the Hounsfield scale.

The study inclusion criteria were encompassed:

osteoporotic patients with traumatic injuries of the lumbar spine and thoracolumbar junction (types A3, A4, B2, and C according to the AOSpine classification) and with neurological symptoms of the C, D, and E severity (according to the ASIA scale);

patients with degenerative spinal stenosis and clinically significant segment instability, who had low back pain and lower extremities pain with a score exceeding 40 (of 100) and a disability index of 40% (according to the Oswestry questionnaire). The radiological criteria for segment instability included sagittal plane translation of >3 mm and angular rotation of >10 degrees [10].

The study exclusion criteria encompassed:

patients with grade III, IV spondylolisthesis;

patients with degenerative deformity, with impaired sagittal balance (SVA >5 cm, PI–LL >10), requiring spinal pelvic fixation or extended fixation of more than 5 segments;

patients with signs of infringement of pedicle screw fixation technique and inadequate bone augmentation;

patients with competing spinal diseases: neoplasms or inflammatory conditions.

Preoperatively, patients were examined for their neurological status and the pain score (using the visual analogue scale, VAS). In patients with degenerative spinal pathology, the disability index (by the Oswestry questionnaire) was determined. Before the operation, all patients underwent CT examination of the lumbar spine; during the study, the radiodensity of the cancellous bone was determined at the standard level L_2_ or L_3_. In patients with traumatic pathology, the measurement was focused on an intact vertebra.

Patients followed up for 18 months (n=153) were divided into three groups:

group 1 (n=39, 16 men and 23 women; mean age 61 (31–79) years) — patients who underwent pedicle screw fixation with bone tissue augmentation with PMMA-based cement. Degenerative pathologies were observed in 6 cases (15.4%), traumatic injuries — in 33 cases (84.6%);

group 2 (n=21, 7 men, 14 women; mean age 63 (39–78) years) — patients who underwent pedicle screw fixation using the proposed technology of vertebral body augmentation with auto- or allograft. There were 5 patients with degenerative pathologies (23.8%) and 16 with traumatic injuries (76.2%);

group 3 (n=93, 38 men, 55 women; mean age 58 (42–81) years) — patients who underwent pedicle screw fixation without bone augmentation. There were 23 cases of degenerative pathologies (24.7%) and 70 (75.3%) — with traumatic pathologies.

All patients with traumatic spinal injuries underwent pedicle screw fixation using intermediate fixation. When the vertebral body height was reduced by >50%, anterior interbody fusion was performed. In the presence of neurological symptoms, we used either ventral or dorsal access for spinal decompression; in the anterior substrate localization, we performed anterior decompression using reconstruction of the anterior column. In all cases, patients with degenerative spinal stenosis underwent microsurgical root decompression and transforaminal interbody fusion.

When performing bone augmentation using PMMA, the vertebroplastic technique was used. In this case, after identifying the vertebral pedicles, vertebroplasty needles were transpedicularly inserted and then extended to the middle of the vertebral body in the sagittal projection. After that, 2 ml of PMMA-based bone cement was injected into the vertebral bodies at each level on each side, after which pedicle screws were applied; then the fixation system was mounted.

When using our original technology of bone tissue augmentation with bone chips, bone funnels were introduced through pedicles into vertebral bodies and then the latter were filled with allo- or autobone chips. As a result, a hyperdense area was created on the trajectory of the subsequent screw placement; then, the bone chips were impacted into the vertebral pedicles. Finally, the screws were transpedicularly inserted on the vertebral bodies, and the transpedicular system was mounted.

In the postoperative period, patients were followed up for 3, 6, and 12 months and examined using the VAS and the Oswestry Disability Index assessment. In addition, spondylograms were taken at 3 months, and spinal CT — at 6 and 12 months. Cases with signs of implant instability were registered. The following radiological criteria of instability were considered: dissociation or breakage of fixator components, bone resorption of >1 mm around the screw, or the formation of a double halo sign — a radiolucent zone around the screw surrounded by a sclerosis zone [11].

### Statistical data analysis

The two-sided Fisher’s exact test was used to assess the difference in the complication rates between the groups, and logistic regression analysis was used to assess the relationship between the complication rate and the surgical technique used. When evaluating the results of statistical analysis, the critical level of statistical significance was p=0.05. Statistical analysis was performed using the Statistica 12.0 software (StatSoft, USA).

## Results

During the observation period, in patients who underwent augmentation using PMMA (group 1), X-ray signs of fixation instability were detected in 11 patients (28.2%); of those, fixator loosening was found in 9 patients, and fixator component breakage — in 2 patients. Notably, the screw loosening occurred in the vertebral pedicles, i.e. there was a proximal loosening. Surgical revisions were conducted in 8 patients (20.5%).

In patients of group 2, who underwent augmentation using the proposed technique (auto- or allograft bone materials), fixator instability was detected in 2 cases. Only one of these two patients required revision: in this case, the destabilization developed according to the well-known windshield wiper effect.

In patients with no bone augmentation (group 3, control), X-ray signs of pedicle screw fixator destabilization were noted in 43 cases (46.2%). Of those, only 15 patients (16.1%) had clinically significant implant destabilization, which necessitated surgical revisions. In all cases, the pedicle screw fixator destabilization was due to screw loosening.

When comparing the complication rates between the groups, it was found that the PMMA-based augmentation significantly reduced the incidence of screw loosening as evidenced by CT scans (p=0.0185, two-sided Fisher’s exact test). However, considering all types of fixator instability, i.e. both loosening and breakage, the incidence rate of implant destabilization (according to CT data) did not significantly differ from the control group (p=0.0801). Yet, the use of augmentation based on autologous and allogeneic bone chips led to a statistically significant decrease in the incidence of implant destabilization (p=0.0023).

The heterogeneity of the enrolled groups might increase data variations: e.g. osteoporotic patients with both degenerative pathology and traumatic injuries were included in the same group. To provide statistical support to the data based on the group mean values, a logistic regression analysis was performed. In the regression model, the incidence of implant destabilization was plotted against the type of degenerative pathology, PMMA-based augmentation, or allograft/autologous bone augmentation. As a result, a statistically significant regression model was obtained: χ^2^=17.9220; p=0.0005 (see the Table).

**Table:** Regression model parameters

Regression equation component	Coefficient and its statistical significance	Odds ratio	
		Value	95% CI
**Intercept**	**0.5941**	**—**	**—**
	**p=0.0437**		
**Degenerative pathology**	**–0.8278**	**–0.4370**	**0.2086–0.9156**
	**p=0.0285**		
PMMA augmentation	0.0484	1.6233	0.6828–3.8597
	p=0.2708		
**Augmentation with allograft or autologous bone**	**1.9973**	**7.3694**	**1.5812–34.3463**
	**p=0.0113**		

The results provide the evidence that degenerative spinal pathology is a risk factor for the development of fixator instability. Augmentation based on allogeneic or autologous bone chips statistically significantly reduces the incidence of this instability, while augmentation with PMMA does not. The relatively low number of revisions did not allow us to determine the effect of the augmentation technique on the incidence of clinically significant destabilization of the pedicle screw fixator.

## Discussion

The increasing elderly population and practicing the urban lifestyle contribute to the higher incidence of degenerative spinal diseases and traumatic injuries that require surgical treatment using pedicle fixation and fusion [1, 2, 12]. In addition, in elderly people, there is also a high frequency of altered bone density that contribute to implant instability and pseudoarthrosis; these factors demand a bone density assessment before surgical treatment [13]. For preoperative examination of patients with degenerative pathologies, spine CT scan is indicated. In this test, it is also possible to measure the radiodensity of the cancellous tissue, which indicates the density of calcium in the bone [5, 13]. According to the current standards, the bone density value of <110 HU corresponds to osteoporosis [13, 14]. Since dual-energy the X-ray absorptiometry (densitometry) data might be overestimated due to summation of bone densities of the vertebral pedicles, facet joints, and vertebral bodies, we used the CT-based radiodensity data as an inclusion criterion for this study.

To increase the strength of screw fixation in bone tissue, various strategies have been developed, including the use of implants with optimal properties, expanding screws, and various augmentation methods. In biomechanical tests for pullout strength [6, 15], the maximal stability was reached by using PMMA augmentation; however, the inherent mechanism of fixator loosening was shown to cause pedicle screw instability in few patients only. In contrast, the fatigue test reproduced the mechanism of fixator loosening in most patients — in this test, stability of PMMA augmented screws was significantly lower [15, 16].

A significant disadvantage of using PMMA is the limited possibility of vertebral pedicle augmentation. In the PMMA technique, the pivot point of the screw is shifted ventrally into the vertebral body [17]. The limited stability of the augmented screws detected in the fatigue test and the biomechanical changes explain the proximal loosening observed after PMMA augmentation [18]. The results showed that PMMA augmentation did not completely solve the problem of pedicle screw instability development, since a significant number of proximal loosening cases became apparent. In addition, this technique does not prevent a fatigue-associated fixator fracture. Therefore, the present results support the conclusion that PMMA augmentation reduces the incidence rate of screw loosening, but does not provide a statistically significant reduction in the total incidence of fixator destabilization.

The aforementioned biomechanical drawbacks of PMMA augmentation, the risk of material leakage into the spinal canal, the extravertebral cement drainage with of a risk of pulmonary embolism, as well as the limited biocompatibility of PMMA (which may manifest in the bone cement implantation syndrome), necessitated the development of an alternative augmentation technique. This novel technique would make it possible to perform, among other things, vertebral pedicle augmentation using a material with better biological compatibility. Bone chips were suggested as a material for vertebral augmentation, but the early results [19, 20] indicated that this technique was less efficient than the PMMA-based approach.

In the course of the present study, we modified this method of augmentation and developed a technique that allowed us to create a hyperdense area that was much larger than the screw diameter; this area could extend from the cranial to the caudal endplate [21]. The proposed method provided a statistically significant decrease in the incidence of implant destabilization and pointed to clear biomechanical advantages of the developed technique. Considering the heterogeneity of the studied groups, we performed a regression data analysis; the results provided more evidence that the proposed technique was more beneficial for pedicle screw fixator stability than the PMMA method.

### Study limitation

These results are preliminary in nature and do not allow assessing the clinical efficacy of the developed method due to the low number of surgical revisions prompted by fixator instability. To assess the full-scale clinical effect, it is necessary to perform a prospective study with the inclusion of a larger number of patients. Nevertheless, the present results allow us to conclude that the developed method significantly reduces the incidence of fixator destabilization and may have an advantage over the PMMA-based augmentation.

## Conclusion

The developed method for vertebral augmentation using bone chips provides a statistically significant decrease in the incidence of pedicle screw fixator destabilization as evidenced by CT data. Due to its biomechanical advantages, the proposed method is potentially more effective in patients with impaired bone density than that based on polymethyl methacrylate.
